# A Prospective Controlled Trial to Evaluate Safety and Efficacy of *in vitro* Expanded Recipient Regulatory T Cell Therapy and Tocilizumab Together With Donor Bone Marrow Infusion in HLA-Mismatched Living Donor Kidney Transplant Recipients (Trex001)

**DOI:** 10.3389/fmed.2020.634260

**Published:** 2021-01-27

**Authors:** Rainer Oberbauer, Matthias Edinger, Gabriela Berlakovich, Peter Kalhs, Nina Worel, Georg Heinze, Michael Wolzt, Thomas Lion, Thomas Wekerle

**Affiliations:** ^1^Division of Nephrology and Dialysis, Department of Internal Medicine III, Medical University of Vienna, Vienna, Austria; ^2^University Hospital Regensburg, Department of Internal Medicine III & Regensburg Center for Interventional Immunology (RCI), Regensburg, Germany; ^3^Division of Transplantation, Department of Surgery, Medical University of Vienna, Vienna, Austria; ^4^Bone Marrow Transplant Unit, Department of Internal Medicine I, Medical University of Vienna, Vienna, Austria; ^5^Department of Blood Group Serology and Transfusion Medicine, Medical University of Vienna, Vienna, Austria; ^6^Section for Clinical Biometrics, Center for Medical Statistics, Informatics and Intelligent Systems, Medical University of Vienna, Vienna, Austria; ^7^Clinical Trials Coordination Centre, Medical University of Vienna, Vienna, Austria; ^8^St. Anna Children's Cancer Research Institute, Vienna, Austria; ^9^Ludwig Boltzmann Institute for Hematology & Oncology, Medical University of Vienna, Vienna, Austria; ^10^Labdia Labordiagnostik GmbH, Vienna, Austria; ^11^Department of Pediatrics, Medical University of Vienna, Vienna, Austria; ^12^Section of Transplantation Immunology, Division of Transplantation, Department of Surgery, Medical University of Vienna, Vienna, Austria

**Keywords:** kidney transplantation, cell therapy, regulatory T cells, chimerism, tolerance, bone marrow, belatacept, tocilizumab

## Abstract

**Background:** The induction of donor-specific immunological tolerance could improve outcome after kidney transplantation. However, no tolerance protocol is available for routine clinical use. Chimerism-based regimens hold promise, but their widespread application is impeded in part by unresolved safety issues. This study tests the hypothesis that therapy with polyclonal recipient regulatory T cells (Tregs) and anti-IL6R (tocilizumab) leads to transient chimerism and achieves pro-tolerogenic immunomodulation in kidney transplant recipients also receiving donor bone marrow (BM) without myelosuppressive conditioning of the recipient.

**Methods/design:** A prospective, open-label, controlled, single-center, phase I/IIa academic study is performed in HLA-mismatched living donor kidney transplant recipients.

**Study group:** Recipients of the study group receive *in vitro* expanded recipient Tregs and a donor bone marrow cell infusion within 3 days after transplantation and tocilizumab for the first 3 weeks post-transplant. In addition they are treated with thymoglobulin, belatacept, sirolimus, and steroids as immunosuppression. Starting 6 months post-transplant, sirolimus and steroids are withdrawn in a step-wise manner in stable patients.

**Control group:** Recipients of the control group are treated with thymoglobulin, belatacept, sirolimus, and steroids as immunosuppression. **Co-primary endpoints** of safety (impaired graft function [eGFR <35 mL/min/1.73 m^2^], graft-vs.-host disease or patient death by 12 months) and efficacy (total leukocyte donor chimerism within 28 days post-transplant) are assessed. **Secondary endpoints** include frequency of biopsy-proven acute rejection episodes and subclinical rejection episodes on surveillance biopsies, assessment of kidney graft function, and the evaluation whether the study protocol leads to detectable changes in the immune system indicative of pro-tolerogenic immune modulation.

**Discussion:** The results of this trial will provide evidence whether treatment with recipient Tregs and donor BM is feasible, safe and efficacious in leading to transient chimerism. If successful, this combination cell therapy has the potential to become a novel treatment option for immunomodulation in organ transplantation without the toxicities associated with myelosuppressive recipient conditioning.

**Trial registration:** European Clinical Trials Database EudraCT Nr 2018-003142-16 and clinicaltrials.gov NCT03867617.

## Introduction

Long-term outcome after kidney transplantation has improved little over the last decades and immune-mediated injury remains a leading cause of graft loss despite modern immunosuppressive drug therapy (1). The establishment of donor-specific immunological tolerance would be a solution to this problem (2, 3). Chimerism-based tolerance, established through the co-transplantation of hematopoietic stem cells together with a kidney from the same donor, has emerged from extensive preclinical research as a promising approach for clinical translation (3, 4). Chimerism was originally observed to lead to tolerance toward the hematopoietic cell donor in naturally occurring freemartin cattle that exchange hematopoietic cells intrauterinely through a shared placental circulation (5). Subsequently, Medawar and colleagues actively induced transplant tolerance by transferring donor hematopoietic cells (6). Over the ensuing decades, the mechanisms contributing to donor-specific tolerance have been delineated in increasing detail. Intrathymic clonal deletion of emerging donor-reactive thymocytes is a key feature of chimerism-based tolerance (7, 8) and one reason why tolerance induced through chimerism typically is very robust. Extrathymic clonal deletion of mature donor-reactive T cells also plays a role, at least in some chimerism protocols (9). Notably, regulatory mechanisms have more recently been recognized as essential mechanisms in chimerism-based tolerance (10, 11). The interplay of deletional and regulatory mechanisms seems to lead to the most durable states of tolerance in pre-clinical chimerism models (4). A cardinal feature of chimerism-based tolerance is its successful translation to large animal models, including non-human primates (3). Besides, anectodal cases of patients that had undergone hematopoietic stem cell transplantation for a conventional indication and that subsequently accepted a kidney graft from the original stem cell donor without immunosuppression provided proof-of-concept that chimerism can also lead to tolerance in humans (2).

Three pilot trials have recently been conducted that investigated the simultaneous co-transplantation of donor hematopoietic stem cells (donor BM or mobilized peripheral blood stem cells [mPBSC]) for the purpose of tolerance induction in living donor kidney transplant recipients (12–16). Tolerance was achieved in 40–60% of HLA-mismatched transplants in two trials and the rate of tolerance was even higher in the HLA-identical kidney transplant setting (13). The protocols of the three trials differ in important aspects, but all have in common that they involve myelosuppressive conditioning (i.e., irradiation of the recipient and/or cytotoxic drug treatment). Total lymphoid irradiation (10 × 80–120 cGy) was part of the Stanford protocol (13), 700 cGy thymic irradiation together with cyclophosphamide was given in the MGH protocol (12) and 200 cGy total body irradiation together with cyclophosphamide and fludarabine in the Northwestern protocol (14). Myelosuppression led to transient leukopenia [absolute neutrophil count <500/μL for ≈12 days in the two HLA-mismatched cohorts (MGH and Northwestern) (12, 14)] and was associated with infectious complications in some cases (17). An engraftment syndrome that was linked to recovery of recipient hematopoiesis following myelosuppression, occurred in all patients in the MGH trial (12). Myelosuppression is considered problematic by many clinicians for use outside of clinical trials in specialized centers. Graft-vs.-host disease (GVHD) occurred in the Northwestern trial (but not the other two trials), leading to one fatality (17). GVHD is widely viewed to be an unacceptable complication in living-donation kidney transplantation and needs to be strictly avoided. Collectively, these landmark trials provide proof-of-concept that chimerism-based tolerance can be achieved in the clinical setting, but routine application of the current protocols in kidney transplant recipients is impeded by safety issues.

A key challenge in the field therefore is to develop a chimerism-based tolerance protocol that is sufficiently safe for more extensive clinical application, but still efficacious. Pre-clinical and clinical data indicate that very low levels of chimerism can suffice for tolerance induction. Besides, chimerism does not need to be permanent but can be transient (12, 18), which can be achieved with milder conditioning regimens. In a series of rodent studies, costimulation blockade (9), mTOR inhibition (19), and IL6 blockade (20) were identified as treatments that allowed conditioning requirements to be reduced. Myelosuppression, however, remained an indispensable factor for achieving engraftment of clinically obtainable BM doses with such protocols. In contrast, the administration of polyclonal recipient Tregs promoted engraftment of fully mismatched BM in the absence of myelosuppression (10). Hematopoietic stem cell engraftment (tested by secondary BM transplantation [BMT]) was achieved across full MHC-barriers in recipients otherwise conditioned only with a short-course of costimulation blockade and the mTOR inhibitor rapamycin. This effect was observed with several types of Tregs (i.e., *in vitro* activated CD4^+^CD25^+^ Tregs, TGFβ-induced Tregs and FoxP3-transduced Tregs) (10). Levels of multi-lineage chimerism were low, but the state of tolerance observed was more complete compared to previous myelosuppressive rodent regimens (achieving higher chimerism levels) with regard to several endpoints (21–23). Recently, the BM engraftment-promoting effect of polyclonal recipient Tregs has been confirmed in a small non-human primate (NHP) study (24), in which Treg application extended overall chimerism and led to chimerism within the T cell lineage. Treg cell therapy is currently explored as promising therapy in several immunological disorders, including the prevention of transplant rejection (25–27) and the prevention of GVHD (28–30). Preliminary results indicate that Treg therapy induced tolerance in a cohort of living-donor liver transplant recipients (31), but the same therapy failed in kidney transplantation (32). The full potential of Treg therapy alone for inducing tolerance in organ transplantation thus still needs to be determined.

**Figure 1:**
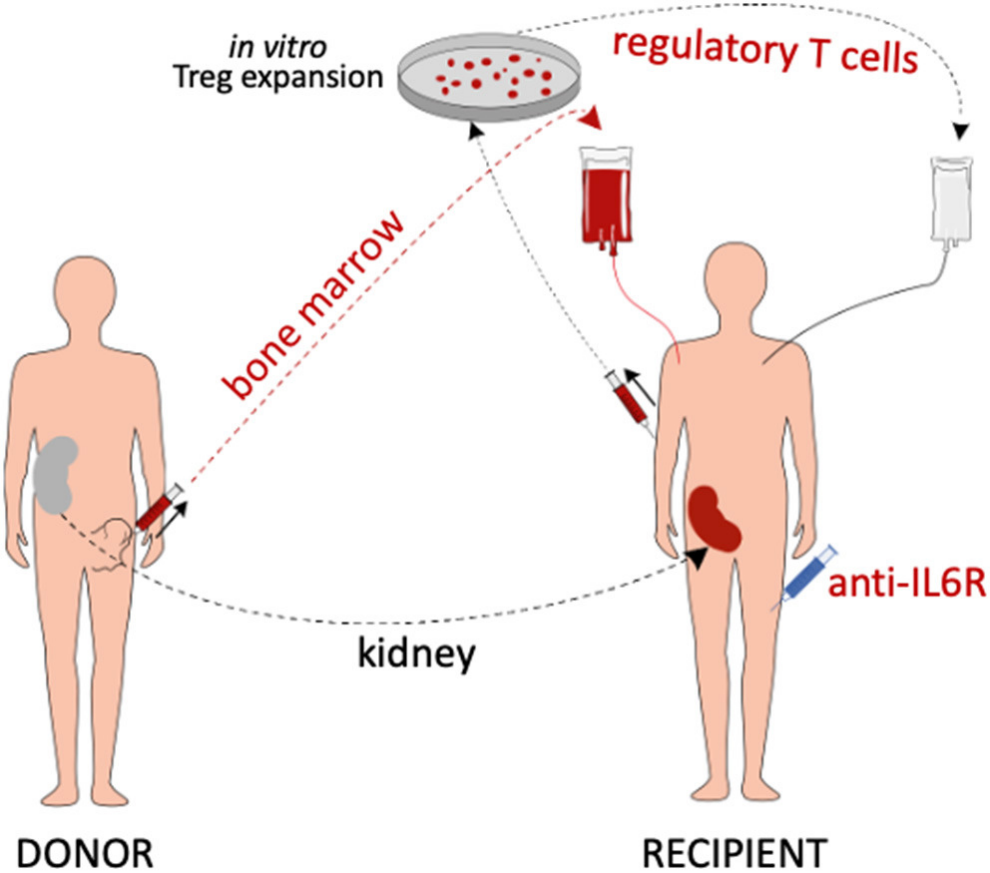
Summary of the study concept. Living donor kidney transplant recipients are treated with *in vitro* expanded polyclonal recipient Tregs, donor BM cells and anti-IL6R mAb (tocilizumab). In addition, they receive belatacept-based immunosuppression (not depicted).

The present study investigates combination cell therapy with recipient Tregs and donor BM, together with IL6 pathway blockade, as a potential strategy for inducing transient chimerism and pro-tolerogenic immunomodulation in kidney transplantation (the study concept is summarized in Figure 1).

## Methods and Analysis

### Protocol Version

Version 2.0; December 4, 2018.

### Trial Sponsor and Role of Sponsor

The Department of Surgery, Medical University of Vienna (Spitalgasse 23, 1090 Vienna, Austria) is the trial sponsor. The study sponsor has no role in the design of the study or the analysis and publication of its results.

### Ethics Approval

The study has been approved by the ethics committee of the Medical University of Vienna (Ethikkommission Medizinische Universität Wien, EK Nr: 1871/2018) and the Austrian Federal Office for Safety in Health Care (BASG Bundesamt für Sicherheit im Gesundheitswesen, Verfahrensnummer 11337515).

### Trial Design

Prospective, open-label, controlled, single-center, phase I/IIa study.

### Subject Population

Subjects are recruited from patients scheduled to receive a living donor kidney transplant at the Vienna General Hospital/Medical University of Vienna.

### Eligibility Criteria

#### Inclusion and Exclusion Criteria

The donor and the recipient need to provide written informed consent. The inclusion and exclusion criteria that need to be fulfilled for enrolment into the trial are the same for recipients in the study group and recipients in the control group. Inclusion/exclusion criteria for donors in the study group are distinct from those for donors in the control group. Inclusion and exclusion criteria are listed in Table 1.

**Table 1:** Inclusion and exclusion criteria for recipients and donors of the study and the control group.

**Inclusion criteria**
For recipients (study and control group):
• Patient has provided written informed consent
• Patient is 18 years or older
• Patient is a planned recipient of a living donor kidney transplant
• Patient is a planned recipient of an ABO blood group-compatible kidney graft
• Patient is a planned recipient of a kidney graft from a donor that is **not** HLA-identical
• Patient is negative for DSA
• WOCBP must have a negative pregnancy test at inclusion
• WOCBP must be using an adequate method of contraception to avoid pregnancy throughout the study and for up to 12 weeks after the study in such a manner that the risk of pregnancy is minimized
For donors (study group only):
• Participant has provided written informed consent
• Participant is 18 years or older
• Participant is suitable to donate bone marrow according to the guidelines of the Department of Blood Group Serology and Transfusion Medicine
• WOCBP must not be pregnant at inclusion (i.e., negative pregnancy test)
For donors (control group only):
• Participant has provided written informed consent to donate blood for immune monitoring analyses
• Participant is 18 years or older
**Exclusion criteria**
For recipients (study and control group):
• Patient is EBV-negative on serology
• Patient is HIV positive or suffering from chronic viral hepatitis
• Patient is CMV negative and receiving a kidney from a CMV-positive donor
• Positive T-cell lymphocytotoxic cross match
• Patient with prior kidney transplant or non-renal solid organ transplant
• Patient has a known contraindication to any of the protocol-specified treatments
• Patient had been diagnosed with a malignancy within 5 years prior to study entry, excluding non-metastatic basal or squamous cell carcinoma of the skin
• Female patients who are breast-feeding
• Female patients with a positive pregnancy test at the time of evaluation

### Interventions

#### Study Group Treatment

**Figure 2:**
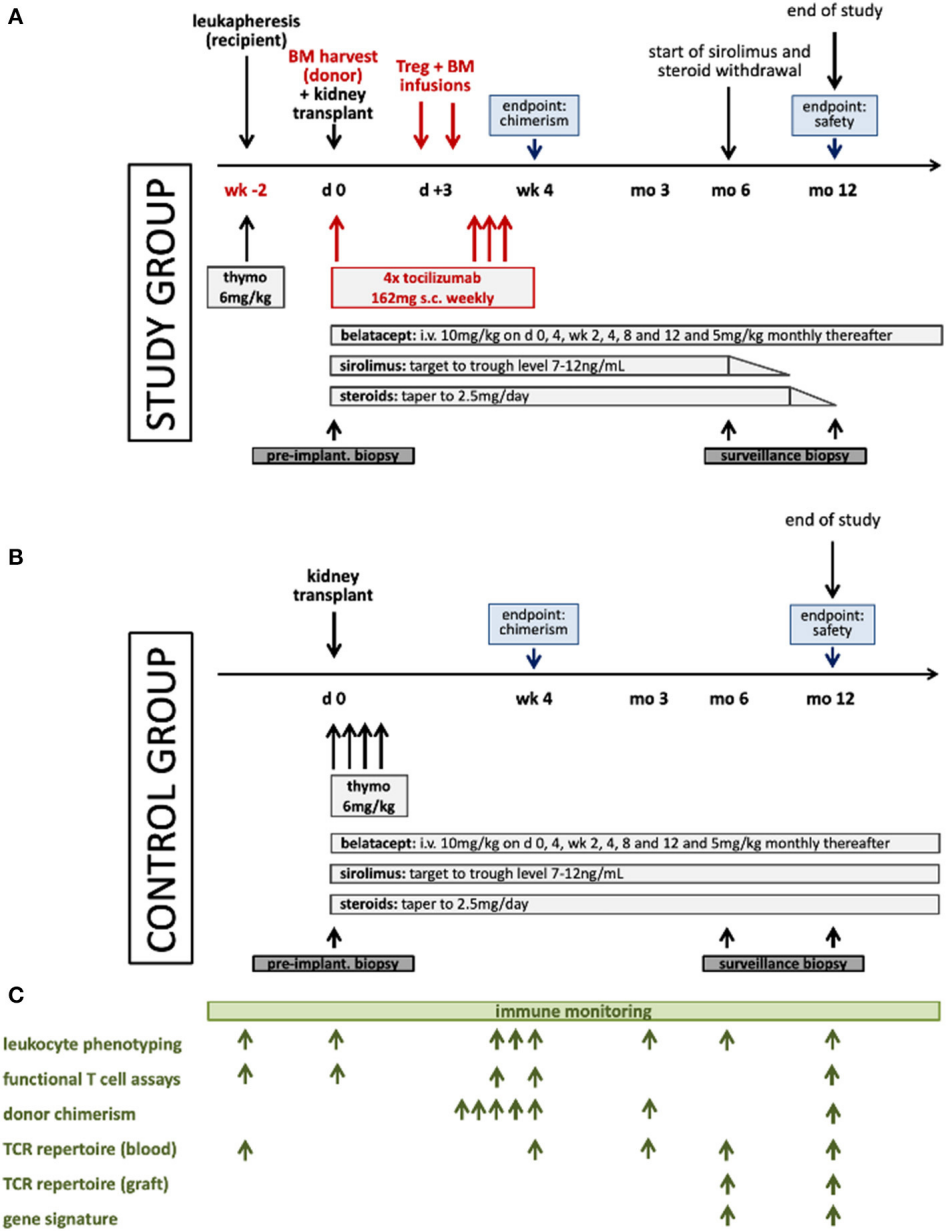
Schematic illustration of the treatment protocols for the study **(A)** and the control **(B)** groups and the immune monitoring plan **(C)**. Thymo denotes thymoglobulin.

The study group receives *in vitro* expanded polyclonal recipient Tregs (RegTivex), anti-IL6R (tocilizumab), and donor BM, in addition to belatacept-based immunosuppression (Figure 2A). Tregs and BM are administered within 3 days after kidney transplantation. Anti-IL6R antibody (tocilizumab) is administered weekly for the first 3 weeks post-transplant. In addition, patients receive immunosuppressive drug therapy consisting of induction therapy with anti-thymocyte globulin (Thymoglobulin) and maintenance therapy with belatacept, sirolimus, and steroids. Starting 6 months post-transplant, sirolimus and steroids are withdrawn in a step-wise manner in stable patients. Sirolimus weaning starts first, to be followed by weaning of steroids 3 months later. By 12 months, stable patients are receiving belatacept monotherapy. *In vitro* expanded regulatory T cells (RegTivex) and tocilizumab (RoActemra; anti-IL6R monoclonal antibody) are the investigational medicinal products (IMPs) of this study.

#### Control Group Treatment

The control group receives the same immunosuppressive drug combination as the study group consisting of thymoglobulin, belatacept, sirolimus, and steroids (Figure 2B). No Tregs and no anti-IL6R are given and no BM is administered. No withdrawal of sirolimus or steroids is attempted.

#### Immune Monitoring

Extensive immune monitoring accompanies the study with the aim to yield information about the activity and the mode of action of the treatment protocol (Figure 2C). Samples (PBMC, serum and DNA) are collected from donors and recipients of both groups at pre-defined time points and are also biobanked for future analysis. A range of laboratory assays have been developed for use in transplant patients. These assays can provide important scientific information on the efficacy of immunomodulatory protocols, but they have to be considered experimental and in large parts are not validated or approved for clinical diagnostics. Results from these assays are not used for clinical decisions. Immune monitoring is planned to include the assessment of lineage-specific chimerism in FACS-sorted cell subsets using the Droplet Digital (dd) PCR technology, leukocyte phenotyping by multi-color flow cytometry, functional T cell assays (IFNγ-ELISPOT, T cell proliferation, and T cell suppressive function), next generation sequencing (NGS)-based T cell receptor (TCR) repertoire analysis of the Treg product and of recipient T cells in blood and in the graft, and intragraft gene expression analysis.

#### Randomization

No randomization is performed. The complex design which requires recipient leukapheresis plus thymoglobulin treatment 2 weeks prior to transplant and cell therapy with Tregs and BM in the recipient and BM harvesting from the donor in the study group but not the control group, would impede enrolment within a reasonable time frame, if randomization were performed, as it is assumed that more subjects are willing to participate in the control than the study group. Due to the small sample size, randomization would require stratification, as otherwise outcome predictors would likely be unevenly distributed between groups. Due to the number of living donor transplants performed annually at the Medical University of Vienna, stratification would likely make enrollment in this single center study infeasible. Therefore, also no attempt at matching the number of HLA-disparities between the groups is undertaken. Thus, mainly for reasons of feasibility no randomization is performed.

#### Treg Cell Product

The IMP consists of *in vitro* expanded CD45RA^+^CD4^+^CD25^high^CD127^low/neg^ regulatory T cells isolated from a leukapheresis product of the kidney transplant recipient. The Treg product (RegTivex) is generated at the *José-Carreras-Centrum for Somatic Cell Therapy* (JCC; www.carreras-centrum.de) at the University Hospital Regensburg (Germany). Two weeks prior to the scheduled kidney transplant, the recipient undergoes standard leukapheresis at the Medical University of Vienna. The time point of leukapheresis is chosen to allow optimum Treg expansion until the scheduled day of infusion. The leukapheresis product is shipped to the JCC using validated transport conditions, enriched for CD25^+^ cells using bead selection technologies, then stained for CD4, CD25, CD127, and CD45RA and FACS-sorted for the CD45RA-positive Treg subpopulation (33). Cells are then *in vitro* expanded for ~2 weeks (2–3 log expansion rates) using CD3/28 stimulation beads and high-dose rh-IL-2 (34). After bead-removal and quality-controlled product release, the Treg cell product is shipped back to Vienna. The cell product is infused within 48 h after release (*note:* Regensburg is a 4-h drive away from Vienna). Kidney transplant recipients of the study group are infused intravenously with the fresh Treg product within 3 days after the kidney transplant. The Treg and BM cell infusions are both given within 3 days post-transplant, but are administered sequentially with a time interval in-between to assess immediate infusion-related side effects individually. At this time, all kidney transplant recipients are routinely treated in-hospital at the transplant center. Treg therapy in this trial constitutes an autologous cell infusion.

The target dose for the Treg cell product is 1.0 × 10^7^ cells/kg body weight. This is the dose for which preliminary safety data exist from GVHD studies. The actual dose of available Tregs/kg body weight will depend on several factors, including the cell number and composition of the leukapheresis product, the efficiency of the Treg expansion and the weight of the recipient. In case the target cell dose cannot be reached, doses as low as 0.3 × 10^7^ cells/kg body weight are permissible. This lower limit is set to minimize the risk that Treg cells from the recipient are discarded without clinical use. The maximum dose of Tregs administered is 1.5 × 10^7^ cells/kg body weight. The cells are administered post-operatively as a single intravenous infusion, no later than 3 days after the kidney transplant (no later than D3).

#### Donor Bone Marrow

Unseparated donor BM cells are used as they were shown to have advantages over mPBSC in pre-clinical experiments and as they were used successfully in a clinical pilot trial of chimerism-based tolerance (12, 15). The target dose is 2–3 × 10^8^ nucleated cells/kg body weight (corresponding to a CD34^+^ cell dose of 2–3 × 10^6^/kg body weight). Similar doses have been used previously in kidney transplant recipients (12, 15). In case of a minor ABO-incompatibility, the BM is plasma-depleted. The unseparated donor bone marrow cells are administered post-operatively as a single intravenous infusion, no later than 3 days after the kidney transplant (no later than D3). Donor bone marrow is only infused in patients that have successfully received a Treg infusion.

#### Tocilizumab

Tocilizumab is administered as four subcutaneous injections of 162 mg each, starting on the day of transplant (D0) and subsequent doses on D5, D15, and D21 post-transplant. This dosing regimen corresponds to the dosing frequency approved for long-term maintenance therapy in rheumatoid arthritis.

#### Anti-thymocyte Globulin of Rabbit Origin (Thymoglobulin)

Thymoglobulin is administered intravenously at a dose of 6 mg/kg body weight. In the control group, thymoglobulin is given at the time of transplant, in four divided doses, as previously reported (35). In the study group, the thymoglobulin administration needs to be moved to an earlier time point, so that thymoglobulin does not deplete the infused Tregs. Besides, it can only be administered after leukapheresis (as the leukapheresis product would otherwise be depleted of the T cell subset necessary for Treg expansion). Therefore, thymoglobulin is administered immediately following leukapheresis, 2 weeks prior to the kidney transplant. Thereby, serum levels of active thymoglobulin (i.e., the fraction that binds to lymphocytes) are low at the time of Treg infusion (36), while numbers of lymphocytes (including T cells) are depleted profoundly. Two weeks after infusion of 6 mg/kg thymoglobulin the level of active thymoglobulin is expected to have fallen to or near the subtherapeutic level (<1 μg/mL) (36), while CD4 T cells are depleted by >95% at this time point (36). A reduction in the number of recipient T cells is considered beneficial or even necessary for the efficacy (activity) of Treg therapy (1). Moreover, the depletion of recipient T cells promotes BM engraftment in rodent and non-human primate studies.

#### Belatacept

Belatacept, a CTLA4Ig derivative, is the only clinically approved alternative to calcineurin-inhibitors (CNIs) as primary immunosuppressant. CNIs impeded outcome in pre-clinical costimulation blockade-based chimerism models (19). Belatacept/CTLA4Ig, in contrast, were associated with pro-tolerogenic mechanisms and promoted BM engraftment in rodent and NHP studies (18). CTLA4Ig was also part of the pre-clinical myelosuppression-free Treg-induced chimerism protocol on which this clinical trial is based (10). The mechanistic interaction of belatacept with Tregs is complex and incompletely understood. Several papers suggest that belatacept might negatively interfere with endogenous Tregs. However, other lines of evidence, including clinical data show a neutral or even favorable effect of belatacept on Tregs (37, 38). The clinically approved dosing regimen of belatacept is used. Belatacept is given as intravenous infusions at a dose of 10 mg/kg on D0, D4, D13, D27, week 8 and 12 and at a dose of 5 mg/kg every 4 weeks thereafter.

#### Sirolimus

mTOR inhibition through sirolimus promotes Tregs while inhibiting effector T cells. In addition, sirolimus promoted the engraftment of allogeneic BM in preclinical studies (10, 19, 39). Sirolimus is dosed to maintain trough levels of 7–12 ng/mL, as in Ferguson et al. (35).

#### Steroids

Corticosteroids (prednisone, prednisolone, methylprednisolone, dexamethasone) are dosed according to local practice at the Medical University of Vienna for 12 months in the control group and 9 months in the study group.

#### BM Harvest

Donor BM (not more than 20 mL/kg body weight; maximum volume 1,500 mL) is obtained from the iliac crest under general anesthesia during the donor nephrectomy surgery (D0). Intraoperative BM harvesting during living donor nephrectomy was reported to be feasible and safe by several groups (40–43).

#### Sirolimus and Steroid Withdrawal in Study Group

Starting 6 months post-transplant, sirolimus and steroids are withdrawn in a step-wise manner in stable patients of the study group. Pre-defined criteria for starting sirolimus and steroid withdrawal are: absence of immunological injury on 6-month surveillance biopsy (i.e., freedom from T cell-mediated rejection and antibody-mediated rejection; borderline score acceptable) and absence of donor-specific antibodies (DSA). Sirolimus weaning starts first, to be followed by weaning of steroids 3 months later.

#### Study Drug Interruption or Discontinuation

The Investigator must temporarily interrupt or permanently discontinue the study drug if continued administration of the study drug is believed to be contrary to the best interests of the patient. The interruption or premature discontinuation of study drug might be triggered by an Adverse Event (AE), a diagnostic or therapeutic procedure, an abnormal assessment (e.g., laboratory abnormalities), or for administrative reasons, in particular withdrawal of the patient's consent. The reason for study drug interruption or premature permanent discontinuation must be documented in the CRF.

### Outcome

Primary Objective:

- 1) To examine the safety of Treg therapy together with tocilizumab and donor bone marrow in living donor kidney transplant recipients.
- 2) To assess chimerism levels within the first month post-transplant.

Secondary Objectives:

- To demonstrate that the study protocol allows the initiation of a step-wise reduction of immunosuppression up to a point when patients receive drug monotherapy.
- To gain insight as to whether the study protocol leads to detectable changes in the immune system indicative of pro-tolerogenic immune modulation.
- To assess the frequency of biopsy-proven acute rejection episodes.
- To assess the frequency of subclinical rejection episodes on surveillance biopsies.
- To assess kidney graft function.
- To assess the area-under-the-curve (AUC) of chimerism.

The composite safety endpoint is defined as the incidence of GVHD, impaired graft function [eGFR <35 mL/min/1.73 m^2^] or patient death, whichever occurs first, within the first 12 months post-transplantation (co-primary endpoint 1).

The efficacy endpoint is defined as individual peak chimerism levels observed in each patient within 4 weeks post-transplant (co-primary endpoint 2). Total leukocyte donor chimerism will be measured by sensitive ddPCR technology at days 5, 10, 15, 21, and 28 post-transplant in both groups (44).

### Sample Size

Six patients (recipients) per group eligible for per-protocol analysis, up to 10 patients per group maximum. For each recipient one donor is enrolled in the same group.

The sample size is based primarily on feasibility to allow enrollment within a reasonable time frame. Beyond this premise, sample size calculation yielded a number of six for the study group to conclude with a probability (statistical power) of 0.8 that the incidence of the safety endpoint is not >65% (**co-primary endpoint 1**) at a one-sided significance level of 2.5% (one-sided test for binomial proportion). This margin of 65% is accepted for statistical analysis to preserve feasibility. A more robust safety analysis will only be possible in potential larger subsequent trials. By including also six patients in the control group the study has 86.5% power to detect a difference in mean peak chimerism on the logarithmic-base-2 scale of 2 log-units (a 4-fold difference in geometric means) given an assumed standard deviation of 1 log-unit (**co-primary endpoint 2**). No data are available from which a meaningful assumption on the standard deviation of mean peak chimerism (on the logarithmic-base-2 scale) could be derived. Our assumption corresponds to a ratio between upper and lower limits of a normal range (97.5th and 2.5th percentiles) of about 16 (four log units), which was considered conservative enough when discussing it among the principal investigators and the study statistician.

### Statistical Methods

#### Analysis Sets

Two different analysis sets are defined:

##### Modified Intention-to-Treat Set

This analysis set includes subjects who were enrolled and received at least one dose of study drug.

##### Per-protocol Set

This analysis set comprises all subjects who received the kidney transplant, the Treg infusion (at a minimum dose of 0.7 × 10^7^ cells/kg body weight), the BM infusion and at least one dose of tocilizumab (study group), all subjects of the control group who were treated with at least one dose of belatacept, and who completed 4 weeks of follow-up and who did not violate the protocol in a way that might affect the evaluation of the effect of the study drug(s) on the primary objective, i.e., without major protocol violations.

#### Endpoints Analysis

##### Primary Endpoint Analysis

*Co-primary Endpoint 1 (Safety)*. Co-primary endpoint 1 will be evaluated in the study group, using the modified intention-to-treat set. The endpoint will be summarized by computing the proportion of patients reaching co-primary endpoint 1 and the corresponding two-sided 95% exact (Clopper-Pearson) confidence interval.

**Null hypothesis:** The incidence of the composite safety endpoint (GVHD, impaired graft function [eGFR <35 mL/min/1.73 m^2^] or patient death by 12 months post-transplant) in treated patients is 65% or higher.

**Alternative hypothesis:** The incidence of the composite safety endpoint (GVHD, impaired graft function [eGFR <35 mL/min/1.73 m^2^] or patient death by 12 months post-transplant) in treated patients is <65%.

For assessing impaired graft function (eGFR <35 mL/min/1.73 m^2^) eGFR values after the first month post-transplant will be considered (to exclude low eGFR values due to delayed graft function in the immediate post-transplant period). eGFR is calculated according to the CKD-EPI (Chronic Kidney Disease Epidemiology Collaboration) formula. GVHD diagnosis needs to be confirmed by histology.

The proportion will be statistically tested against the null hypothesis of the proportion being 65% or greater using a one-sample test for the binomial proportion at a one-sided significance level of 0.025. The null hypothesis will not be rejected (corresponding to a negative study result) if more than one out of the six patients experiences the safety endpoint, as then the upper limit of the one-sided 97.5% confidence interval for the safety endpoint incidence will reach beyond 65%.

*Co-primary Endpoint 2 (Efficacy)*. Total leukocyte donor chimerism is measured by sensitive ddPCR technology on D5, D10, D15, D21, and D 28 post-transplant in both study groups. Co-primary endpoint 2 is defined as the individual peak chimerism level, i.e., the maximum value over the five measurements taken in each patient (C_max_). The endpoint will be evaluated in the per-protocol set.

The endpoint will be summarized by computing mean and standard deviation of log-base-2 peak chimerism in both groups. Mean values of the two treatment arms will be compared using a one-sided two-sample *t*-test assuming unequal variance (Satterthwaite-Welch-test) with a significance level of 0.025.

**Null hypothesis:** The expected level of chimerism (log_2_ C_max_) in patients treated with the experimental treatment is not higher than that of control patients.

**Alternative hypothesis:** The expected level of chimerism (log_2_ C_max_) in patients treated with the experimental treatment is higher than that of control patients.

The two null hypotheses cover different aspects and thus the two co-primary endpoints will be tested independently and both will be reported. Under the global null hypothesis and assuming independence of safety and efficacy, the one-sided probability to reject one or two null hypotheses is 5%.

##### Secondary Endpoint Analysis

All secondary endpoints will be analyzed in a purely descriptive manner. Thus, any inferential measures, such as *p*-values and confidence intervals are only meant to describe the precision of summary statistics, but not to confirm or reject a study hypothesis. Categorical data will be expressed as frequency counts and percentages. Continuous variables will be summarized by their mean, standard deviation, minimum and maximum. For repeatedly measured outcomes, pre- and all post-baseline values will be assessed and graphically presented.

### Oversight and Monitoring

Monitoring is performed by the Clinical Trials Coordination Centre of the Medical University of Vienna. A Data and Safety Monitoring Board (DSMB) that it is independent from the sponsor and has no competing interests regularly reviews the clinical data of the trial and has the authority to recommend alterations and/or termination of the trial for safety reasons.

### Adverse Event Reporting and Harms

An Adverse Event (AE) is defined as any untoward adverse change from the subject's baseline condition, i.e., any unfavorable and unintended sign including an abnormal laboratory finding, symptom or disease which is considered to be clinically relevant by the physician that occurs during the course of the study, whether or not considered related to the study drug. A special section is designated to adverse events in the case report form documenting the details of the AE. The severity of clinical AEs is graded on a three-point scale: mild, moderate, severe, and reported on specific AE pages of the CRF.

For all AEs, the Investigator assesses the causal relationship between the study drug and the AE using his/her clinical expertise and judgment (not related—unlikely—possibly related—probably related—related).

A Serious Adverse Event (SAE) is defined by the International Conference on Harmonization (ICH) guidelines and GCP guidelines as any AE fulfilling at least one of the following criteria:

- Results in deaths.
- Life-threatening—defined as an event in which the subject was, in the judgment of the Investigator, at risk of death at the time of the event.
- Requiring subject's hospitalization or prolongation of existing hospitalization.
- Resulting in persistent or significant disability or incapacity (i.e., a substantial disruption of a person's ability to conduct normal life functions).
- Congenital anomaly or birth defect.
- Is medically significant or requires intervention to prevent at least one of the outcomes listed above.

In case of a serious adverse event, the Investigator has to use all supportive measures for best patient (recipient and donor) treatment. A written report is also to be prepared detailing the SAE. If applicable, the initial report should be followed by a follow up report, indicating the outcome of the SAE.

Suspected unexpected serious adverse reactions (SUSARs) are defined as all serious adverse reactions with suspected causal relationship to the study drug that is unexpected (not previously described in the Summary of Product Characteristics or Investigator's brochure) and serious. The regulatory authorities, and the Institutional Review Board/Independent Ethics Committee (IRB/IEC) must be informed about all SUSAR. Such reports containing all relevant detail shall be made by the sponsor.

### Trial Status

Recruitment started on August 1, 2019 and is expected to be completed by April 2022.

## Discussion

Several considerations informed the trial design. The trial is designed with patient safety as highest priority. There is no myelosuppression, and thus no engraftment syndrome is expected (which is related to recovering recipient hematopoiesis after myelosuppression). The risk of GVHD is minimal as it is expected that no permanent chimerism is induced. Immunosuppression is reduced to belatacept monotherapy in the study group, but complete withdrawal is not attempted in this first trial of combined Treg and BM cell therapy. Thereby the potential risk of immunological damage to the graft during immunosuppression withdrawal is avoided (15, 40). Unseparated donor BM at approximately the proposed dose (i.e., ≈3 × 10^8^ nucleated cells/kg body weight) has been used safely (without GVHD) in several clinical transplant trials of “BM augmentation” (i.e., BM infusion without myelosuppressive conditioning) (41, 42, 45, 46). Moreover, it has been demonstrated that donor BM cell infusion does not lead to sensitization (i.e., no *de novo* DSA) in patients receiving belatacept-based immunosuppression (with sirolimus and alemtuzumab) (42).

While a control group was not included in the three pilot trials of chimerism-based tolerance published to date (12–14), its inclusion in the study design reported herein allows more robust conclusions to be drawn from the study. Due to the complex design which requires recipient leukapheresis and BM harvesting from the donor in the study group but not the control group, no randomization will be performed. A non-experimental immunosuppressive regimen, for which clinical data from a phase II trial exist, was selected for use in the control group, in order to minimize any risk for patients in this group and to allow an overall favorable risk-benefit assessment of the study. Therefore, also no BM infusion is given in the control group. This group will serve as reference for assessing the primary and secondary endpoints and for comparison of immune monitoring results. Since BM infusion alone (i.e., without concomitant myelosuppression or Treg therapy) was associated only with very low levels of chimerism (41, 45, 46) or no detectable chimerism (42) in kidney recipients, the control group provides an important reference for comparing chimerism levels (co-primary endpoint 2).

The protocol is designed to achieve transient chimerism without myelosuppression. Pre-clinical data demonstrate that the administration of polycloncal recipient Tregs at the time of BM infusion can achieve BM engraftment in this setting. The mechanisms how this effect is mediated are not fully delineated yet, but control of donor-reactive NK cells appears to be critical (47). Blocking the IL6 pathway was also shown to promote BM engraftment in the absence of myelosuppression, albeit with less potency (20). This effect is associated with an increase in the frequency of endogenous Tregs. Therefore, in the present trial Treg therapy and tocilizumab are given at the time of BM infusion within the shortest time frame considered clinically safe. BM infusion should be given at or near the time of kidney transplantation as delayed administration is less successful and required additional conditioning in non-human primate studies (48).

The *in vitro* expansion of the recipient Tregs takes 2–3 weeks (34). At the time of protocol submission the cell product was only approved for administration as fresh (i.e., unfrozen) product. Therefore, leukapheresis is timed to take place 2 weeks before the scheduled kidney transplant, so that after the expansion the fresh Treg cell product can be administered immediately after kidney transplant. If frozen Treg products are available, the leukapheresis of the recipient could be performed at earlier time points and the cell product could be stored until the kidney transplant.

Thymoglobulin is included in the study protocol for two reasons: (1) belatacept universally requires induction therapy, with basiliximab having been used in the original phase II and III trials. Thymoglobulin has subsequently shown promise as induction agent in combination with belatacept in a phase II trial, in which low rates of rejection were observed (35); and (2) thymoglobulin-mediated T cell depletion is considered to increase the therapeutic efficacy of transferred Tregs (1) which then encounter reduced numbers of T cells/lymphocytes. Besides, recipient T cell depletion promotes BM engraftment. Notably, as thymoglobulin is a polyclonal antibody preparation it targets a wide range of surface molecules that are not only expressed on T cells, but also on other leukocyte subsets (49), and thus potentially depletes also other effector lymphocytes, thereby possibly further promoting BM engraftment.

The decision to wean patients to belatacept monotherapy instead of sirolimus monotherapy was informed by several considerations, but especially the experience with belatacept monotherapy in other trials (42) and the relatively high risk of sirolimus discontinuation due to side effects.

While the small sample size is a limitation of the trial, it will be sufficient to allow a conclusion as to whether such a combination cell therapy regimen is feasible in principle and whether this concept induces transient chimerism without myelosuppression. Together with the insight from the immune monitoring assays the results of the trial are expected to permit an informed decision as to whether a subsequent trial testing Treg therapy together with donor BM infusion should be conducted and how its design should look like.

The combination of Treg therapy and donor BM infusion has shown considerable promise in pre-clinical models. The present Trex001 trial translates this novel strategy to the clinical setting. It is expected the trial will yield valuable data regarding the potential of this approach, which eventually could become a new immunomodulatory therapy in kidney transplantation with the ultimate goal of improving long-term outcome.

## Ethics Statement

The studies involving human participants were reviewed and approved by the ethics committee of the Medical University of Vienna (Ethikkommission Medizinische Universität Wien, EK Nr: 1871/2018) and the Austrian Federal Office for Safety in Health Care (BASG Bundesamt für Sicherheit im Gesundheitswesen, Verfahrensnummer 11337515). The patients/participants provided their written informed consent to participate in this study.

## Author Contributions

RO, ME, GB, PK, NW, GH, MW, TL, and TW designed the study protocol. TW developed the underlying study concept and wrote the draft of the manuscript. All authors approved the final version of the manuscript.

## Conflict of Interest

The authors declare that the research was conducted in the absence of any commercial or financial relationships that could be construed as a potential conflict of interest.
